# Clinical effects of Chemotherapy combined with Immunotherapy in patients with advanced NSCLC and the effect on their nutritional status and immune function

**DOI:** 10.12669/pjms.39.2.6365

**Published:** 2023

**Authors:** Jin Jiao, Wen-wen Li, Yan-hong Shang, Xiao-fang Li, Meng Jiao

**Affiliations:** 1 Jin Jiao, Department of Oncology, Affiliated Hospital of Hebei University; Hebei Provincial Key Laboratory of Tumor Radiochemotherapy Mechanism and Procedure Research, Baoding, Hebei 071000, P.R. China; 2 Wen-wen Li, Department of Oncology, Affiliated Hospital of Hebei University; Hebei Provincial Key Laboratory of Tumor Radiochemotherapy Mechanism and Procedure Research, Baoding, Hebei 071000, P.R. China; 3 Yan-hong Shang, Department of Oncology, Affiliated Hospital of Hebei University, Baoding, Hebei 071000, P.R. China; 4 Xiao-fang Li, Department of Oncology, Affiliated Hospital of Hebei University, Baoding, Hebei 071000, P.R. China; 5 Meng Jiao, Department of Oncology, Affiliated Hospital of Hebei University, Baoding, Hebei 071000, P.R. China

**Keywords:** Advanced NSCLC, Chemotherapy, Immunotherapy, Immune function, Nutritional status

## Abstract

**Objectives:**

To evaluate the clinical effects of chemotherapy combined with immunotherapy in patients with advanced non-small-cell lung cancer (NSCLC) and the effect on their nutritional status and immune function.

**Methods:**

Total 120 patients with advanced NSCLC admitted to Affiliated Hospital of Hebei University from May 2019 to October 2021 were randomly divided into two groups (n= 60, respectively). Patients in the control group were treated by chemotherapy with cisplatin-paclitaxel (TP) alone: 120 mg/m^2^ paclitaxel was used on d1; and 25mg/m^2^ cisplatin (CDDP) was used for more than two hour, once every 14 days, for three consecutive three cycles. Patients in the study group were additionally given 200 mg sindilizumab by intravenous drip, once every three weeks. The contrastive analysis of clinical effects, the incidence of adverse reactions, improvement of the nutrient index and the changes in levels of CD3^+^, CD4^+^, CD8^+^, and CD4^+^/CD8^+^ in T-lymphocyte subsets was performed between the two groups.

**Result:**

The overall response rate (ORR) was 80% and 61% in the study group and the control group, respectively; and the difference was statistically significant (p=0.03); the contrast analysis of the incidence of post-treatment adverse drug reactions (ADRs) in patients in the two groups suggested that the incidence of adverse reactions was 33.3% and 45% in the study group and the control group, respectively; and the difference was not statistically significant (p=0.19). After the treatment, the improvement of hemoglobin, albumin, serum iron and ferritin levels in the study group was more significant than that in the control group; and the difference was statistically significant (p < 0.05). After the treatment, the levels of CD3^+^, CD4^+^ and CD4^+^/CD8^+^ in the study group were much higher than those in the control group; and the difference was statistically significant (p < 0.05).

**Conclusion:**

Chemotherapy combined with immunotherapy is effective in treating patients with advanced NSCLC without increasing the incidence of adverse reactions, and can significantly improve their nutritional status and T-lymphocyte function. This therapeutic regimen is of much higher clinical value than the chemotherapy-only regimen.

## INTRODUCTION

The data from the epidemiological survey showed that^1^ lung cancer is still the malignant tumor with the highest morbidity and mortality worldwide. Non-small cell lung cancer (NSCLC) accounts for more than 85%.^2^ What’s worse, more than 70% of patients with NSCLC are in an advanced stage when diagnosed and cannot receive operative treatment.^3^ The risk factors of NSCLC include smoking, environmental factors and genetic factors. NSCLC has no specific clinical manifestations in the early stage. Therefore, most of the confirmed cases found in clinical work are in an advanced stage, which imposes great impacts on patients’ health and life.^4^ As a common palliative therapy for patients with advanced NSCLC, chemotherapy realizes clinical treatment by killing or inhibiting tumor cells with chemotherapy drugs. Clinically, patients with NSCLC are often treated by platinum-based chemotherapy regimens. However, there is a high risk of recurrence after the treatment.^5^ Moreover, patients receive chemotherapy repeatedly, which reduces the immune function of the body and increases relevant adverse reactions. Meanwhile, the nutrient depletion status of tumors also leads to the abnormal nutritional status of patients and lower treatment tolerance, which ultimately results in the reduction of the efficacy of chemotherapy.^6^

The emergence of immunotherapy has completely changed the situation of advanced NSCLC. Multiple clinical trials have proved the safety and feasibility of the combination of chemotherapy and immunotherapy.^7^ The programmed cell death receptor-1 (PD-1) and its ligand of immune checkpoint proteins can interact with anti-PD-1 antibodies, improving the objective response rate of cancer patients. Sindilizumab is a humanized monoclonal antibody against PD-1 and can be used for the treatment of recurrent or refractory tumors after second-line systemic chemotherapy.^8^ The combination of TP chemotherapy with sindilizumab immunotherapy had good clinical effects in patients with advanced NSCLC.

Our objective was to evaluate the clinical effects of chemotherapy combined with immunotherapy in patients with advanced non-small-cell lung cancer (NSCLC)

## METHODS

One hundred twenty patients with advanced NSCLC admitted to our hospital from May, 2019 to October, 2021 were selected and randomly divided into two groups (n = 60, respectively). There were 38 male and 22 female patients aged 46~77 (average 62.47±11.92 years) in the study group. There were 35 male and 25 female patients aged 45~77 (average 62.08±10.97 years) in the control group. There was no significant difference in the general data of patients between the two groups. However, there still was comparability between the two groups (Table-I). The study was approved by the Institutional Ethics Committee of Affiliated Hospital of Hebei University (No.:2019Q054; dated: 1^st^ March, 2019), and written informed consent was obtained from all participants.

**Table-I:** Contrastive Analysis of General Data Between the Study Group and the Control Group (*χ̅*±*S*) n=60

Index	Study group	Control group	t/χ^2^	P
Age (y)	62.47±11.92	62.08±10.97	0.19	0.85
Male (%)	38 (65%)	35 (62.5%)	0.31	0.57
Clinical stage			0.21	0.65
III	47 (70%)	49 (75%)		
IV	13 (30%)	11 (25%)		
Tumor location			0.14	0.71
Peripheral	38 (67.5%)	36 (62.5%)		
Central	22 (32.5%)	24 (37.5%)		
Pathological type				
Adenocarcinoma	32 (57.5%)	35 (55%)	0.30	0.58
Squamous cell carcinoma	16 (35%)	14 (40%)	0.18	0.67
Miscellaneous	12 (7.5%)	11 (5%)	0.05	0.82

P > 0.05

### Inclusion criteria:

- Patients who met the diagnostic criteria for advanced NSCLC;^9^
- Patients with chest imaging (CT or MRI) showing the presence of lesions that can be accurately measured;^10^
- Patients who were in a good physical condition instead of a dependent state (KPS>80);^11^
- Patients between 40 and 77 years of age;
- Patients and their families who had good compliance with treatment and were willing and able to cooperate with the completion of this study;
- Patients who had no contraindications for drugs used in this study;
- Patients who had signed the informed consent.

### Exclusion Criteria:

- Patients with the poor general condition and unstable vital signs;
- Patient complicated with other systemic malignancy;
- Patients complicated with severe organic disease;
- Patients who were allergic or intolerant to any drug involved in this study;
- Patients who were unable to cooperate to complete this study;
- Patients who had taken any drug that affects the study, such as immunosuppressor and hormone.

### Therapies

After admission, patients in both groups completed relevant laboratory examinations, including blood cell analysis, coagulation function, liver function and kidney function. Patients with abnormal indexes were treated accordingly. Hydration was performed one day before chemotherapy, patients in the control group were treated with TP regimen alone, specifically as follows: 120 mg/m^2^ paclitaxel on d1; 25 mg/m^2^ CDDP, for more than two hours, tested during chemotherapy, with antiemetic, liver and kidney function protection, rehydration and other therapies applied, once every 14 days, for three consecutive cycles.^12^ Patients in the study group were additionally given 200 mg sindilizumab by intravenous drip, once every three weeks.^13^

### Observation Indicators: Efficacy evaluation:

Tumor efficacy evaluation was performed once every two cycles after treatment; and patients were observed for three consecutive months. *Methods of clinical efficacy determination^14^:* complete remission (CR): The lesions disappear completely and the tumor markers return to normal for more than four weeks, which including carcinoembryonic antigen (CEA), neuron specific enolase (NSE) and cytokeratin 19 serum fragment 21-1 (CYFRA21-1); partial remission (PR): The volume of lesions decreases by more than 30% for more than four weeks; stable (SD): The volume of lesions decreases by < 30% or increases by < 30%; progress (PD): The volume of lesions increases by more than 30% or new lesions appear; overall response rate (RR) = CR+PR%; *Evaluation of ADRs:* ADRs occurred in both groups within one month after medication, including fever, bone marrow suppression, gastrointestinal reactions, liver and kidney dysfunction and other adverse reactions, were recorded; *Improvement of nutritional status:* The fasting blood in the morning was sampled before and after the treatment, respectively; and the changes in such nutritional indexes as hemoglobin, albumin, serum iron and ferritin before and after the treatment were compared and analyzed. 4) *Analysis of immune status:* The fasting blood in the morning was sampled before and after the treatment respectively to detect the levels of CD3^+^, CD4^+^, CD8^+^ and CD4^+^/CD8^+^ in T-lymphocyte subsets; and the contrastive analysis of the differences before and after the treatment between the two groups was performed.

### Statistical Analysis:

The software SPSS 20.0 was used for the statistical analysis of all data. The measurement data were expressed as (±S). Independent samples t-test was used for the data analysis between the two groups. Paired t-test was applied to intra-group data analysis. χ^2^ test was used for rate comparison. A *p*-value of <0.05 was considered statistically significant.

## RESULTS

The contrast analysis of the effects between the two groups is shown in Table-II. It suggested that the ORR was 80% in the study group and 61% in the control group. The effects in the study group were evidently better than those in the control group, and the difference was statistically significant (p=0.03).

**Table-II:** Contrastive Analysis of Efficacy between Two Groups (*χ̅*±*S*) n=60.

Group	CR	PR	SD	PD	ORR
Study group	23	25	7	5	48 (80%)
Control group	21	16	14	9	37 (61%)
c^2^					4.88
P					0.03

P < 0.05.

The contras analysis of the incidence of ADRs after the treatment between the two groups suggested that the incidence of adverse reactions was 33.3% in the study group and 45% in the control group; the difference was not statistically significant (p=0.19). (Table-III)

**Table-III:** Contrastive Analysis of ADRs after Treatment between Two Groups (*χ̅*±*S*) n=60.

Group	Fever	Bone marrow suppression	Liver dysfunction	Kidney dysfunction	Gastrointestinal reactions	Incidence
Study group	5	3	4	5	3	20 (33.3%)
Control group	6	6	4	7	4	27 (45%)
c^2^						1.71
P						0.19

p > 0.05

After the treatment, the levels of nutritional indexes including hemoglobin, albumin, serum iron and ferritin in the study group and the control group were higher than those before the treatment, which indicated that patients’ nutritional status was improved after chemotherapy; the improvement in the study group was greater than that in the control group; and the difference was statistically significant (p=0.00) (Table-IV).

**Table-IV:** Contrastive Analysis of Serum Nutritional Indexes before and after Treatment Between Two Groups (*χ̅*±*S*) n=60.

Group	Hemoglobin (g/L)*	Albumin (g/L)*	Serum iron (mmol/L)*	Ferritin (ug/L)*
Study group	6.68±2.17	5.03±1.75	7.43±2.15	5.42±2.30
Control group	4.36±1.49	3.57±1.38	5.32±2.04	3.19±1.74
t	6.23	5.07	5.51	5.98
p	0.00	0.00	0.00	0.00

* p < 0.05.

There was no significant difference in the pre-treatment levels of CD3^+^, CD4^+^, CD8^+^ and CD4^+^/CD8^+^ between the two groups (P > 0.05). The post-treatment levels of CD3^+^, CD4^+^, CD8^+^ and CD4^+^/CD8^+^ in the study group were significantly higher than those in the control group; and the difference was statistically significant (p < 0.05). However, the changes in the levels of CD8+ were not obvious (p = 0.96) (Table-V).

**Table-V:** Contrastive analysis of levels in T-lymphocyte subsets before and after treatment between two groups (*χ̅*±*S*) n=60.

Index		Study group	Control group	t	p
CD3+ (%)	Pre-treatment	44.73±8.75	44.32±8.25	0.26	0.79
	Post-treatment*	50.31±8.53	46.24±8.07	2.67	0.01
CD4+ (%)	Pre-treatment	25.40±4.51	25.53±4.82	0.15	0.88
	Post-treatment*	38.11±7.39	34.27±7.84	2.62	0.01
CD8+ (%)	Pre-treatment	21.85±4.13	21.75±4.31	0.13	0.89
	Post-treatment	22.62±5.14	22.57±5.07	0.05	0.96
CD4+/CD8+	Pre-treatment	1.38±0.41	1.35±0.52	0.35	0.73
	Post-treatment*	1.96±0.51	1.46±0.27	6.71	0.00

* p < 0.05.

## DISCUSSION

Lung cancer is the malignant tumor with the highest morbidity and mortality in China. Approximately 85% of patients with lung cancer were diagnosed with NSCLC. Since there are no specific clinical symptoms in the early stage, most patients with NSCLC are in an advanced stage when diagnosed. These patients are usually treated by chemotherapy. For some advanced-stage patients, although chemotherapy can improve prognosis and survival rate, adverse reactions are also obvious. The efficacy of chemotherapy in patients with NSCLC in the middle and advanced stages has been in a bottleneck period.^15^ In recent years, the effect of the immune mechanism in tumorigenesis and progression has been clarified. Accordingly, targeted therapy is also becoming clear. Targeted therapy can significantly improve the prognosis of some patients with NSCLC, but some patients treated by targeted therapy have drug resistance problems.^16^ Furthermore, there are no corresponding targeted drugs for patients with NSCLC with negative driver genes^17^, making new therapy a clinical problem to be solved urgently.

Immunotherapy has greatly changed the therapy for newly diagnosed advanced NSCLC. At present, more and more lung cancer diagnosis and treatment guidelines have recommended immune drugs in the treatment of advanced NSCLC, so as to benefit patients.^18^ Immunotherapy combined with chemotherapy has achieved good results and has improved the progression-free survival of these patients.^19^ Proto et al.^20^ held that PD-1 inhibitors combined with platinum-based chemotherapy have become an effective first-line therapy. PD-1 is distributed on the surface of immune cells, while programmed death-1 ligand (PDL-1) is distributed on the surface of tumor cells. The combination of the two results in the immune escape of tumor cells by activating the signal pathway in immune cells.^21^ PD-/PDL-1 blockade can improve the tumor infiltrating lymphocytes (TIL) killing effect.^22^

The combination and improvement of immunotherapy and chemotherapy have significantly improved the prognosis of patients with NSCLC. The immune checkpoint blockade with PD-1 and PD-L1 antibodies can produce long-lasting reactions of clinical significance in patients with advanced NSCLC. The more extensive use of these drugs can improve the nutritional status and survival rate of patients with advanced lung cancer.^23^ Immunotherapy combined with platinum-based chemotherapy is showing increasing benefits in the treatment of patients with advanced NSCLC.^24^ The study of Zhang et al.^25^ proved that this regimen can improve the function of T lymphocytes and restore their anti-tumor effect. Jiang et al.^26^ believed that the combination shows controlled toxicity and encourages anti-tumor activity. The results of a multi-center study showed that^27^: When the combination of chemotherapy and immunotherapy was adopted, only about 20% of patients withdrew from the study due to adverse reactions, while most patients could complete the whole course of treatment and achieve good results. Besides, the study of Leonetti^28^ also proved the significant clinical benefits of immune checkpoint inhibitors combined with chemotherapy in treating NSCLC. This study demonstrated that the ORR of chemotherapy combined with immunotherapy for advanced NSCLC was 80%, while the ORR in the control group was 61% (p=0.03); the incidence of adverse reactions was 33.3% and 45% in the study group and the control group, respectively, and the difference was not statistically significant (p=0.19); the post-treatment levels of CD3^+^, CD4^+^ and CD4^+^/CD8^+^ in the study group increased significantly, and the difference was statistically significant (p < 0.05). The results showed that the cellular immune status of patients was significantly improved after combined immunotherapy and that the clinical effect was significant without significantly increasing adverse reactions.

The common adverse reactions during chemotherapy include gastrointestinal reactions, nutritional deficiencies and impaired immune function^29^ Improving patients’ immune status is of great significance for the nutritional status of cancer patients.^30^ This study also proved that the improvement of the post-treatment levels of hemoglobin, albumin, serum iron and ferritin in the study group was more significant than that in the control group; and the difference was statistically significant.

### Limitations:

It includes small sample size and short follow-up period. In the future clinical work, the sample size and follow-up period will be further increased and the effect of different therapies on the long-term effect and survival of patients further improved, so as to evaluate the benefits of this regimen to patients in a more comprehensive manner.

## CONCLUSION

In conclusion, chemotherapy combined with immunotherapy is effective in treating patients with advanced NSCLC, and can significantly improve their nutritional status and T-lymphocyte function. There was no obvious increase in the incidence of adverse reactions. Therefore, this therapeutic regimen is of much higher clinical value than the chemotherapy-only regimen.

### Authors’ Contributions:

**JJ and WL** designed this study, prepared this manuscript, are responsible and accountable for the accuracy and integrity of the work.

**MJ** collected and analyzed clinical data.

**YS and XL** participated in acquisition, analysis, or interpretation of data and draft the manuscript.
